# A Case of Neglected Posterior Fracture Dislocation of the Shoulder Treated With Greater Tuberosity Osteotomy

**DOI:** 10.1155/2024/6486750

**Published:** 2024-06-11

**Authors:** Masashi Koide, Satoshi Tateda, Sayaka Miyasaka, Akihiro Yasuyama, Yoichi Sasaki, Mika Abe

**Affiliations:** Department of Orthopedic Surgery, Japanese Red Cross Ishinomaki Hospital, Ishinomaki, Japan

## Abstract

Posterior dislocation of the shoulder joint is a rare condition. It is often misdiagnosed owing to a lack of evident clinical features compared with anterior shoulder dislocation, and inappropriate radiological examination. We present a case of chronic posterior fracture dislocation treated with greater tuberosity osteotomy. A 66-year-old man was injured in a fall while carrying a drone. He was referred to our hospital following 3 months of conservative treatment at a nearby clinic, without reduction of the posterior dislocation. Physical examination revealed a prominent reduction in shoulder joint range of motion and shoulder pain. Radiological examination revealed posterior shoulder dislocation associated with greater tuberosity malunion and a small bone fracture of the posterior portion of the glenoid. Open reduction and internal fixation, including greater tuberosity osteotomy, were performed. Although subluxation of the posterior dislocation persisted postoperatively, the humeral head gradually returned to its centric shoulder joint position owing to rotator cuff force coupling. At 24-month follow-up, the patient showed excellent shoulder results.

## 1. Introduction

Posterior dislocation of the shoulder joint is a rare condition that occurs in 2%–5% of all shoulder dislocations [1]. Posterior fracture dislocation is even more rare, reportedly involving 0.9% of 1500 posterior fracture dislocations of the shoulder in 0.6 per 100,000 individuals annually [2–4]. The most common causes include high-energy trauma, seizures, and electrocution [5]. Posterior shoulder dislocation is often misdiagnosed because of a lack of evident clinical features compared with anterior shoulder dislocation and inappropriate radiological examination [6]. It has been reported that 60% of cases of posterior shoulder dislocation are missed by a physician at the initial presentation [7]. In contrast to anterior shoulder dislocation, posterior dislocation does not show striking deformities, and radiographic evaluation is often inappropriate [6]. The anteroposterior view shows a light bulb-like appearance, which is easily misinterpreted as a normal finding. Determining the precise position of the humeral head is challenging if a true scapular Y view is not obtained. An axillary view can be difficult to obtain owing to pain. Loss of external rotation range of motion is a characteristic feature of posterior dislocation; therefore, computed tomography (CT) is recommended in doubtful cases.

A chronic dislocation is considered if the diagnosis is made > 6 weeks after the injury [8]. This type of dislocation is difficult to reduce in a closed manner and requires surgery when a posterior dislocation is present for > 6 weeks [9]. There are many treatment options for posterior fracture dislocation including closed reduction, open reduction and internal fixation, allograft reconstruction, lesser tuberosity/subscapularis transfer, rotational osteotomy, posterior bone block, posterior Bankart repair, a modified McLaughlin procedure, arthroplasty, and conservative treatment [10–14]. A greater tuberosity osteotomy was performed for posterior fracture dislocation of the shoulder in the case presented here for the following three reasons: (i) the humeral head and glenoid bone stock were preserved, (ii) there was no rotator cuff deficiency, and (iii) this was a young and active patient. Few studies have reported the precise technique and treatment progress of greater tuberosity osteotomy for posterior fracture dislocation of the shoulder [8]. This case report describes the use of greater tuberosity osteotomy for posterior fracture dislocation of the shoulder, along with subsequent treatment and management. Written informed consent was obtained from the patient involved in this case report.

## 2. Case Presentation

A 66-year-old right hand-dominant male site manager was referred to our hospital with right shoulder pain and an inability to move his right arm. He had fallen from a step when carrying a drone 3 months previously and had visited a nearby clinic on the day of injury. He was diagnosed with a greater tuberosity fracture and managed conservatively without reduction of the posterior dislocation. Despite healing of the greater tuberosity fracture, his shoulder function did not improve.

Clinical examination revealed a prominent loss of range of motion and pain in the right shoulder. Right-sided shoulder elevation was restricted at 60°, abduction was restricted at 50°, external rotation was −60°, and internal rotation was only possible as far as the buttock level. The humeral head was in a locked internally rotated position, and external rotation was not possible. His Constant–Murley score was eight. Despite the posterior dislocation of the humeral head, the greater tuberosity was in its correct anatomical position relative to the scapula; therefore, the greater tuberosity could be appropriately palpated. Posterior dislocation of his right shoulder joint was irreducible under an interscalene nerve block.

**Figure 1:**
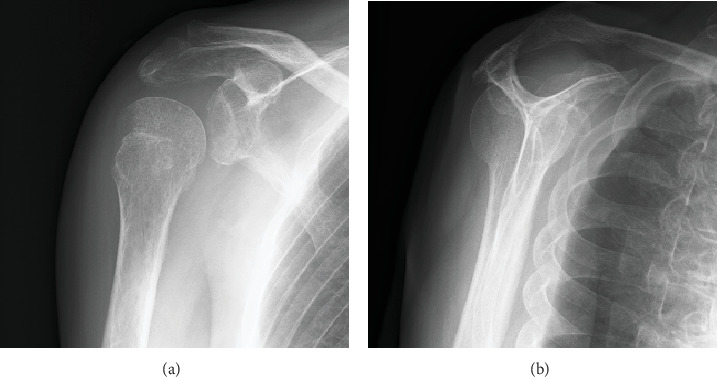
Initial plain radiographic images on first referral to our hospital. (a) An anteroposterior radiograph showing a light bulb-like appearance of the humeral head with a malunited greater tuberosity. (b) A scapular Y view showing the posterior shift of the humeral head.

**Figure 2:**
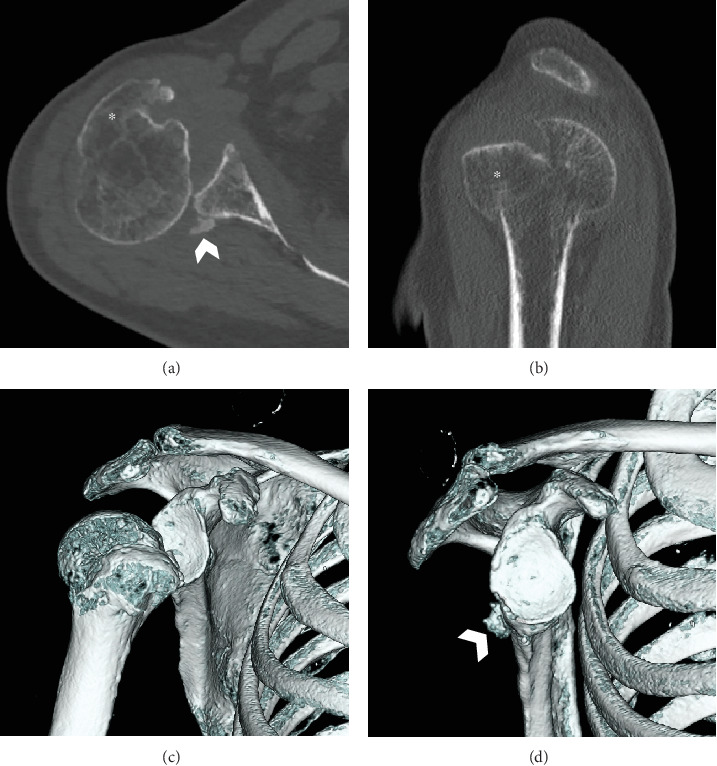
Preoperative CT. (a) Axial and (b) sagittal views showing posterior shoulder dislocation with a malunited greater tuberosity and a small glenoid fracture of the posterior portion. (c, d) 3D reconstruction of the CT images. (d) The humerus was removed to observe the glenoid. Asterisk: greater tuberosity; white arrowhead: glenoid fracture. CT: computed tomography; 3D: three dimensional.

**Figure 3:**
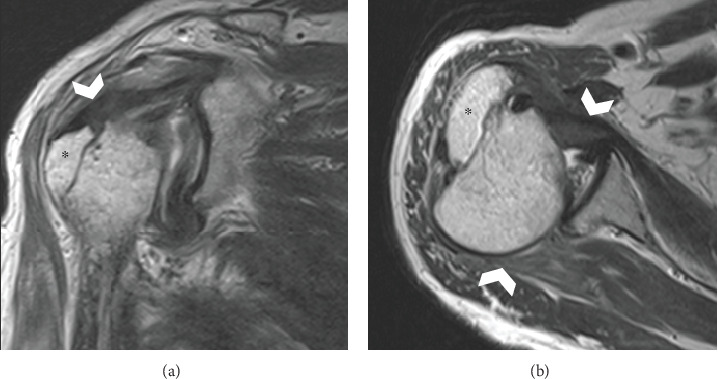
Preoperative MRI. (a) A coronal view showing an intact rotator cuff supraspinatus tendon. (b) The axial view showing an intact rotator cuff and subscapularis and infraspinatus tendons and no dislocation of the long head of the bicep tendon. Asterisk: greater tuberosity; white arrowhead: intact rotator cuff. MRI: magnetic resonance imaging.

Anteroposterior plain radiography revealed a light bulb-like appearance of the humeral head with a malunited fracture of the greater tuberosity (Figure 1(a)). A scapular radiographic Y view showed a posterior shift of the humeral head (Figure 1(b)). CT images showed posterior dislocation with a malunited greater tuberosity and a small bone fracture of the posterior glenoid (Figure 2). A reverse Hill–Sachs lesion was not evident in the anteromedial portion of the humeral head. Magnetic resonance imaging (MRI) revealed an intact rotator cuff; however, the infraspinatus and teres minor tendons appeared to be thin because the humeral head had dislocated posteriorly (Figure 3). No injury to the bicep tendon was observed. We assumed that the original position of the fracture line of the malunited greater tuberosity was just lateral to the intertubercular groove of the long head of the bicep tendon.

**Figure 4:**
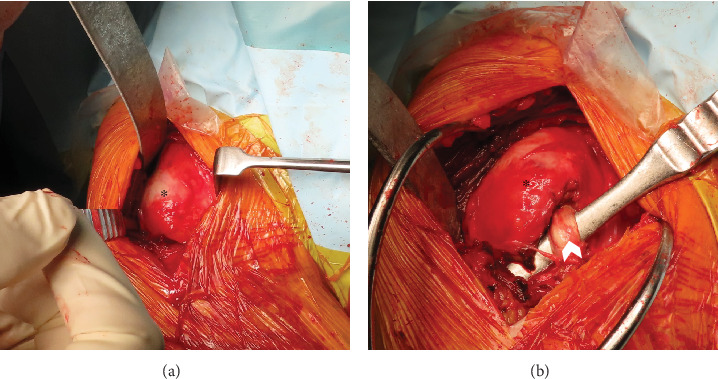
Intraoperative view. (a) A malunited greater tuberosity was observed using the deltopectoral approach. The greater tuberosity protrudes just lateral to the intertubercular groove. (b) Following a release of the groove, the long head of the bicep tendon was observed. A malunited greater tuberosity is easily recognizable. Asterisk: greater tuberosity; white arrowhead: long head of bicep tendon.

**Figure 5:**
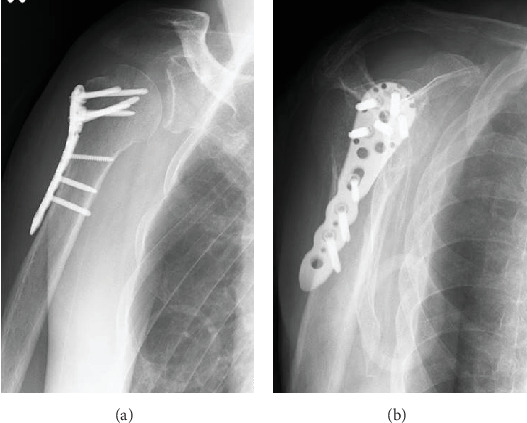
Plain radiographic images on postoperative Day 1. (a) AP view, (b) Y view.

The patient was scheduled for surgery. He was placed in a beach chair position and treated using a combined deltopectoral and miniposterior approach under general anesthesia [15]. After splitting the deltoid and pectoralis major muscles, the proximal humerus was observed. The malunited greater tuberosity protruded lateral to the intertubercular groove (Figure 4(a)). We then attempted a reduction procedure; however, the shoulder joint was irreducible. We opened the groove to reveal the long head of the bicep tendon (Figure 4(b)). Soft tissue tenodesis of the long head of the bicep tendon and a pectoralis major muscle and rotator interval split were conducted to visualize displacement of the malunited greater tuberosity in the lateral groove. We performed an osteotomy of the greater tuberosity using a bone chisel parallel to the protruded anterior fracture line. The posterior dislocation was reduced with insertion of the elevatorium through the rotator interval between the humeral head and the glenoid, and external rotation of the humeral head was then possible. The greater tuberosity was reduced, and internal fixation with a plate (Axsos 3 Ti Proximal Humerus Plating System; Stryker, MI, USA) was performed under external rotation to avoid dislocation posteriorly (Figure 5). A McLaughlin procedure was not performed because a reverse Hill–Sachs lesion was not evident in this case.

We used a miniposterior approach just prior to the reduction of the posterior dislocation to fix the small fracture fragment of the posterior glenoid via a straight 4 cm long incision parallel to the joint line from the acromial angle. After splitting the deltoid muscle, the deep layers of the infraspinatus and teres minor muscles were exposed. The posterior joint capsule was revealed during a blunt procedure in-between the infraspinatus and teres minor muscles. An anchor (Q fix; Smith & Nephew, UK) was inserted into the posterior glenoid fracture site, and the small fracture was sutured following joint capsulotomy. The joint capsule and the space between the infraspinatus and teres minor muscles were firmly sutured after reduction to prevent posterior displacement.

Postoperatively, the arm was braced for 6 weeks using a shoulder orthosis set to 20° abduction and 20° external rotation. Passive forward elevation and external rotation of the shoulder were permitted at 1 week postoperatively. Internal rotation was prohibited until the locking position on the orthosis was released. Active exercises commenced at 8 weeks postoperatively, and strengthening exercises commenced at 3 months postoperatively.

**Figure 6:**
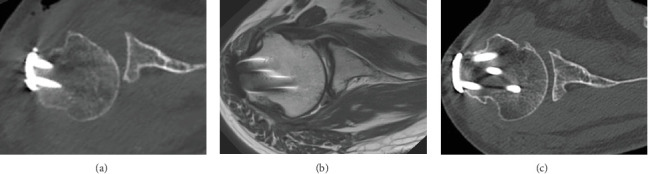
Postoperative CT and MRI examinations. (a) CT on postoperative Day 1. (b) MRI 3 months postoperatively. (c) CT images taken 12 months postoperatively. The centric position of the humeral head showing gradual improvement. CT: computed tomography; MRI: magnetic resonance imaging.

**Figure 7:**
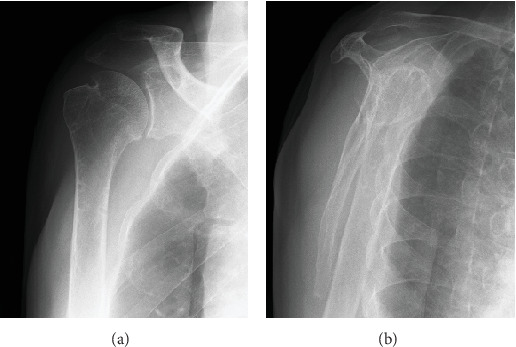
Plain postoperative radiographic images. Plain postoperative radiographic images taken at 24 months postoperatively. (a) AP view, (b) Y view.

**Figure 8:**
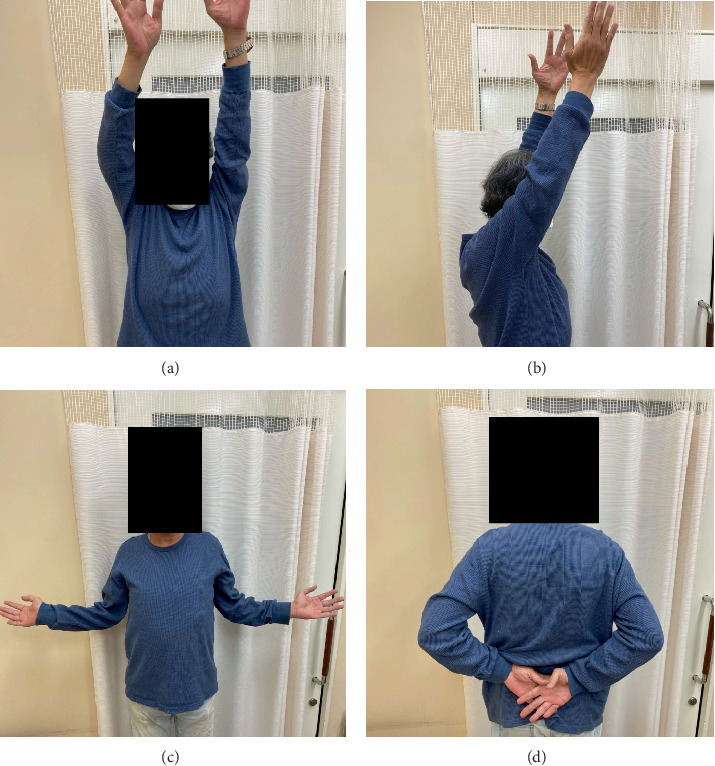
Final follow-up at 24 months postoperatively. (a, b) Active elevation. (c) Active external rotation. (d) Active internal rotation.

A CT scan on postoperative Day 1 showed posterior subluxation of the humeral head (Figure 6(a)). To prevent recurrence of the posterior dislocation, we instructed the patient not to rotate the arm internally past the neutral position, and passive external rotation was encouraged. A 3-month postoperative MRI (Figure 6(b)) and a 12-month postoperative CT scan showed gradual improvement in the centric position of the humeral head (Figure 6(c)). The patient regained a pain-free full range of motion after 12 months, with no signs of instability. At 18 months postoperatively, he underwent implant plate removal (Figure 7). At 24 months postoperatively, his functional status was maintained, no complications were observed (Figure 8), and his Constant–Murley score had improved to 93.

## 3. Discussion

This patient had a chronic posterior dislocation of the shoulder with a malunited fracture of the greater tuberosity. As the posterior dislocation was irreducible due to the malunited greater tuberosity, we performed an osteotomy of the greater tuberosity. Subluxation of the posterior dislocation was observed after surgery; however, the humeral head gradually recovered to the centric position due to force coupling of the rotator cuff.

Treatment options vary for posterior shoulder dislocations depending on the duration of dislocation, the size of the humeral head defect, the severity of the glenoid bony injury, the integrity of the rotator cuff, and the patient's functional demand [3, 14]. With more severe clinical presentations where dislocation is accompanied with extensive damage to the bony structures, cartilage, or ligaments, or with osteoarthritis, shoulder arthroplasty is preferred to reduce the dislocation and optimize long-term shoulder stability and function in older adult patients [16–18]. In contrast, if the degree of instability in the shoulder joint is not severe without bone loss of the humeral head and glenoid fossa, preservation of the shoulder joint should be considered in young active patients. Paparoidamis et al. reported a treatment algorithm for posterior shoulder dislocation based on the duration of the injury, the presence of fracture, and the size of the reverse Hill–Sachs lesion [14]. In this algorithm, open reduction, internal fixation, and stabilization are recommended for patients with a high functional demand and chronic (> 6 weeks postinjury) posterior fracture dislocation of the shoulder in all cases. The extent to which the degree of shoulder joint instability would remain following open reduction and internal fixation for posterior fracture dislocation of the shoulder joint has not been clarified. In our case, although the subluxation of the posterior dislocation was observed after the greater tuberosity osteotomy, the posterior instability gradually improved owing to rotator cuff integrity over approximately 3 months. Greater tuberosity osteotomy is a reasonable treatment choice for posterior dislocations associated with severe malunion of the greater tuberosity in patients with a high functional demand when there is no rotator cuff deficiency. It may be inappropriate to select a greater osteotomy for a posterior fracture dislocation with an irreparable rotator cuff tear even without intra-articular bony structure injury.

The rotator cuff is an important shoulder joint stabilizer [11, 16]. Rotator cuff tears are known to occur in association with traumatic anterior shoulder dislocation; however, their association with posterior shoulder dislocation is not well known. Posterior fracture dislocations show a locked fashion as they are accompanied with simultaneous fractures of the humerus or scapula [19–21]. Bony injuries to the glenoid and humeral head can lock into place, thereby preventing closed reduction [18]. Open reduction and internal fixation should be considered even for chronic conditions if there is no deficiency in terms of rotator cuff integrity. It is important to observe whether external rotation is possible intraoperatively to effectively address the locked condition of the posterior shoulder dislocation and to avoid harming the attachment of the rotator cuff and the greater tuberosity. This is necessary to ensure that rotator cuff function is regained and that the humeral head is repositioned back to the glenoid fossa. Mittal, Jain, and Gamanagatti reported 16 cases involving treatment for chronic posterior shoulder dislocation, with patients undergoing immobilization of the shoulder spica in 45° external rotation for 4 weeks postoperatively [8]. Seo et al. reported an irreducible posterior fracture dislocation of the shoulder with a massive rotator cuff tear due to incarceration of the long head of the bicep tendon in a 57-year-old male patient [16]. They performed arthroscopic in situ superior capsule reconstruction using the long head of the bicep tendon and restricted external rotation motion in the shoulder joint to 30° for 6 weeks postoperatively. In our case, the humeral head returned to the centric position of the shoulder joint due to force coupling of the rotator cuff despite posterior dislocation subluxation having occurred postoperatively. In cases of posterior fracture dislocation with massive rotator cuff tears, reverse arthroplasty is a treatment option for older adult patients.

In conclusion, we described a patient who underwent greater tuberosity osteotomy to treat a chronic posterior fracture dislocation of the shoulder. Release of the locking position in internal rotation and force coupling of the intact rotator cuff were important in this treatment. This treatment option should be selected based on the duration of injury, the size of bone loss in the humeral head and glenoid, the severity of malunion of the greater tuberosity, and the presence of a rotator cuff tear.

## Data Availability

The datasets analyzed during the current study are available from the corresponding author.
